# Fisetin induces autophagy in pancreatic cancer cells via endoplasmic reticulum stress- and mitochondrial stress-dependent pathways

**DOI:** 10.1038/s41419-019-1366-y

**Published:** 2019-02-13

**Authors:** Shengnan Jia, Xiaodong Xu, Senhao Zhou, Yan Chen, Guoping Ding, Liping Cao

**Affiliations:** 1 Department of General Surgery, Sir Run Run Shaw Hospital, School of Medicine, Zhejiang University, 310000 Hangzhou, Zhejiang China; 2 Department of General Surgery, Huzhou Hospital, Zhejiang University School of Medicine, 313003 Huzhou, Zhejiang China; 3 Innovation Center for Minimally Invasive Technique and Device, Zhejiang University, 310000 Hangzhou, Zhejiang China

**Keywords:** Mitophagy, Pancreatic cancer, Chaperone-mediated autophagy, Endoplasmic reticulum

## Abstract

Pancreatic cancer is one of the most aggressive tumors and patients have poor survival rates. Fisetin, a natural flavonoid, was recently reported to have antitumor effects in various cancer models. Autophagy is a conserved catabolic process that maintains cellular homoeostasis in response to stress, and together with apoptosis, determines cell fate. Herein, we examined the effect of fisetin on pancreatic cancer. We reveal that fisetin inhibits PANC-1 cell proliferation using a real-time cell analysis system. Moreover, the in vivo antitumor effect of fisetin was verified in pancreatic cancer using a luciferase-expressing murine xenograft pancreatic cancer model. We found that the AMPK/mTOR signaling pathway was enhanced after fisetin treatment; however, autophagy was not diminished by adding the AMPK inhibitor compound C. Thus, we hypothesized that an another autophagy regulating pathway existed. RNA-seq analysis revealed that the unfolded protein response pathway, which is activated by ER stress, was enriched. We also found that the stress-induced transcription factor p8 was increased in fisetin-treated PANC-1 cells, and that fisetin-induced autophagy was blocked by silencing p8. We revealed that p8-dependent autophagy was AMPK-independent, and that p8 regulated ATF6, ATF4, and PERK in response to ER stress via p53/PKC-α-mediated signaling. Furthermore, mitophagy was associated with Parkin and PINK1 in response to mitochondrial stress. Interestingly, ATF4 and ATF6 were increased in cells treated with fisetin and compound C. Moreover, inhibiting the AMPK/mTOR pathway with compound C may upregulate p8-dependent autophagy. Thus, there may be crosstalk between the AMPK/mTOR and p8-dependent pathways.

## Introduction

Pancreatic cancer, also known as pancreatic ductal adenocarcinoma (PDAC), is one of the most aggressive tumors and leads to high mortality and poor survival rates; the 5-year survival of pancreatic cancer patients is 6% due to early metastasis and chemotherapy resistance^1,2^. As pancreatic cancer patients are mostly symptomless, less than 20% of patients receive a diagnosis early enough for surgical resection^2^. Although the nucleotide analogue gemcitabine is used as the standard chemotherapy for PDAC^3^, some patients receive few benefits as a result of chemoresistance^4^. Thus, novel treatments are urgently needed.

Fisetin (3,7,3′,4′-tetrahydroxyflavone) is a natural flavonoid that is primarily present in vegetables and fruits, such as cucumber, onion, apple and strawberry^5^. Fisetin is known to possess multiple pharmacological activities, such as antioxidant^6^, anti-inflammatory^7^, and anticancer effects in various cell types^8–10^. Fisetin induces apoptosis in colon cancer HCT-116 cells by inhibiting expression of the transcription factor heat shock factor 1^9^. In gastric cancer cells, fisetin causes mitochondria-dependent apoptosis^10^. From these reports, it appears that the antitumor mechanism of fisetin may be cancer-cellspecific. However, there have been few studies focused on the effect of fisetin in PDAC. Murtaza et al. found that fisetin inhibited the growth of pancreatic cancer AsPC-1 cells through death receptor 3 (DR3)-mediated inhibition of the nuclear factor kappa B (NF-κB) pathway^11^.

Autophagy is a catabolic process in which cytoplasmic contents are delivered to lysosomes through double-membrane autophagosomes for bulk degradation. Although autophagy is usually thought of as a process that mitigates various types of cellular stress to promote survival, abnormal autophagy has been implicated in the pathophysiology of cancers, and even results in cancer cell death^12–14^. Furthermore, abnormal autophagy is involved in both cell survival and cell death in pancreatic cancer^15,16^. Depending on the degraded substrate, such as mitochondria, ribosomes, endoplasmic reticulum (ER), peroxisomes, and lipids, autophagy has been divided into mitophagy, ribophagy, reticulophagy, pexophagy and lipophagy, respectively^17–19^. Suh et al. showed that fisetin induces autophagy in prostate cancer by inhibiting the mammalian target of rapamycin (mTOR) pathway^20^. Interestingly, another study showed that fisetin inhibited autophagy and induced caspase-7-associated apoptosis in casepase-3-deficient breast cancer MCF-7 cells^21^. However, only a few studies have focused on fisetin-induced autophagy in cancer cells, and this type of induced autophagy has not been investigated in PDAC. Further studies are needed to determine the role of autophagy in fisetin-treated PDAC cells.

The transcription factor p8, also known as nuclear protein transcriptional regulator 1 (NUPR1), is a transcription cofactor that is strongly induced by different cellular stresses^22–24^. As a critical player in cell stress, p8 has been implicated in several physiological and pathophysiological processes and is associated with autophagy^25,26^. The key sensors of ER stress are inositol-requiring transmembrane kinase and endonuclease 1, activating transcription factors 4 (ATF4) and 6 (ATF6), and protein kinase RNA-like ER kinase (PERK), which are also involved in inducing autophagy upon ER stress^27,28^. PERK activates eIF2α, which in turn regulates ATF4 expression. Our previous results showed that p8 regulates autophagy in response to ER stress via an mTOR-independent pathway, which modulates PERK and ATF6 via activating p53 and protein kinase C-α (PKC-α) signaling^29^.

In this study, we analyzed the inhibition of human pancreatic cancer cell growth and proliferation by fisetin in vitro and in vivo. Our results indicated that autophagy was primarily induced via a p8-dependent pathway that regulated PERK, ATF4, and ATF6 in response to ER stress. Additionally, we found evidence for mitophagy associated with mitochondrial stress in fisetin-treated PANC-1 cells.

## Results

### Fisetin inhibited the viability of human pancreatic cancer cells in vitro and in vivo

**Fig. 1:**
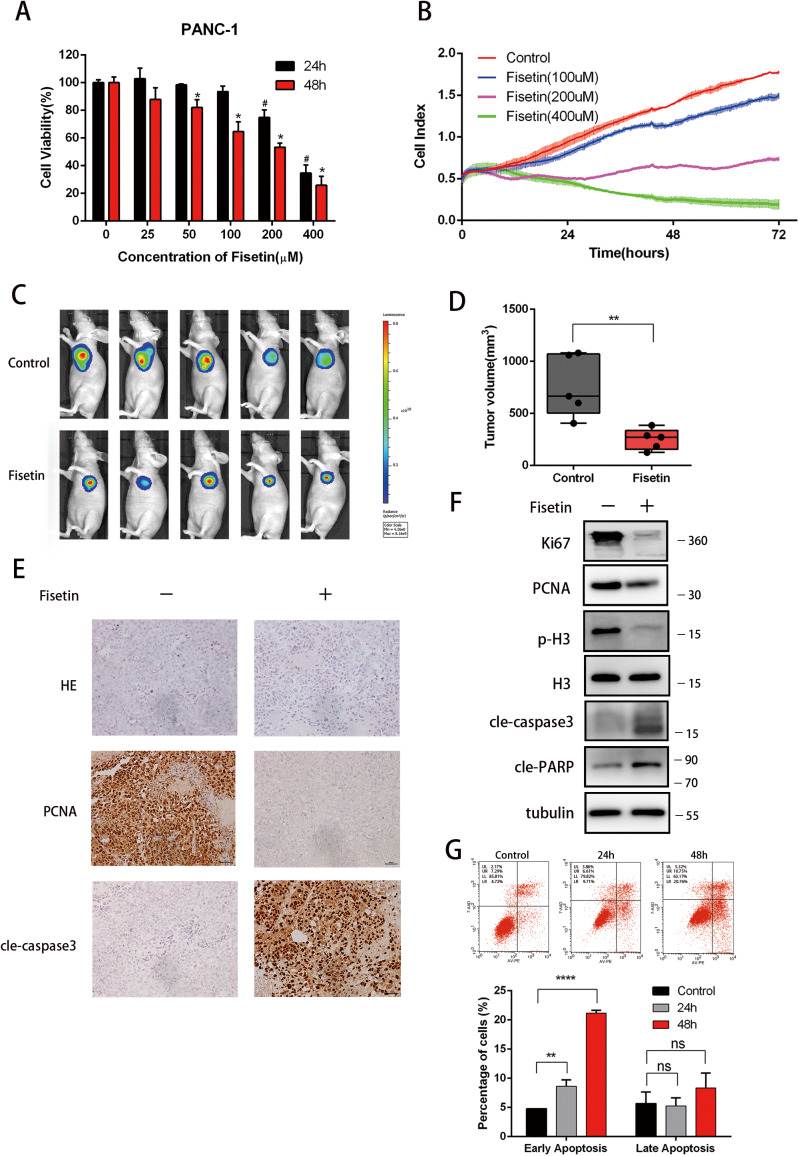
Effect of fisetin on viability of PANC-1 cells in vitro and in vivo **a** Cell viability of PANC-1 cells was measured by CCK-8 assay. Cells were treated with fisetin (0, 25, 50, 100, 200, and 400 μM) for 24 and 48 h, respectively. The absorbance was measured at 450 nm. Data are presented as mean ± SD; *P ≤ 0.05, #*P* ≤ 0.05. **b** Real-time cell analysis (RTCA) of pancreatic cancer PANC-1 cells. Cells were seeded in 96 well Eplates. Then cells were treated with fisetin (0–400 μM)for 72 h. Cell growth was monitored using the xCELLigence RTCA DP Instrument (Roche). Impedance was recorded every 15 min using an xCELLigence system. The times on the *x*-axis indicate the times after treatment. The Cell Index on the *y*-axis reflects a consistent, logarithmic relationship to cell number. Each point represents the mean value from three replicates with SDs. **c** A total of 1 × 10^6^ PANC-1-Luciferase cells were injected into the shoulders via a left subcostal incision. Control group mice were treated with DMSO via intraperitoneal injection, and treatment group mice were treated with Fisetin (300 mg/kg body weight i.p.) every other day. After 20 days treatment, mice imaged for bioluminescence. Data were expressed as average radiance (photons/second/square centimeter/steradian). **d** The differences in tumor volume between control and fisetin treatment groups at 20 days after treatment. Results are mean ± SD (*n* = 5), **P ≤ 0.01. **e** The expression levels of PCNA, cleaved-caspase 3 in control and fisetin treatment groups of mice were detected by immunohistochemistry. Bar scale 50 μm. **f** Western blotting analyses of Ki67, PCNA, p-H3, cleaved-caspase3, and cleaved PARP. **g** Apoptosis of cells with treatment. Cells were treated with fisetin (200 μM) for 24 and 48 h, followed by Annexin-V/PI staining and flow cytometry analysis. Data are presented as mean ± SD; **P ≤ 0.01, ****P ≤ 0.0001, NS no significance

To determine the effect of fisetin on PDAC cells, we treated pancreatic cancer PANC-1 and BxPC-3 cells with increasing concentrations of fisetin (0, 25, 50, 100, 200, and 400 µM), and measured the cell viability with Cell Counting Kit-8 assay (Fig. 1a, Fig. S1A). Interestingly, we found that low concentrations of fisetin (25, 50 µM) did not markedly inhibit the viability of PANC-1 cells efficiently, whilst BxPC-3 cells were sensitive to low concentrations of fisetin. Thus, the differential sensitivity may be cell specific. Moreover, we explored the effect of fisetin in the human normal pancreatic duct cell line hTERT-HPNE. The results showed that fisetin was less cytotoxic to normal cells compared with pancreatic cancer cells (Fig. S1B). To further examine the effect of fisetin on the growth and viability of human pancreatic cancer PANC-1 cells, we used a real-time cell analysis system (xCELLigence, Roche, Basel, Switzerland), which uses label-free microelectronic biosensor technology, to dynamically monitor cytotoxicity and cell death^30,31^. The results are presented as cell index (CI) values, which were measured by the electronic cell sensor array located at the bottom of electronic microtiter tissue culture plates^30^. PANC-1 cells were cultured with increasing concentrations of fisetin (0, 100, 200, and 400 µM), and the results showed that fisetin reduced PANC-1 cell viability in a dose- and time-dependent manner (Fig. 1b). Together, these results showed that fisetin inhibited the proliferation of PANC-1 cells.

Although the number of studies on fisetin have increased over recent years, there have been few investigations into its in vivo antitumor activity; furthermore, there are no published results for the effect of fisetin on PDAC. To determine the in vivo efficacy of fisetin, we used a nude mouse xenograft model of luciferase-expressing human pancreatic PANC-1 tumor cells, which emit bioluminescence after intraperitoneal luciferin injection. This allowed the noninvasive detection of tumor size and position in living mice. Notably, tumor sizes were significantly reduced in fisetin-treated mice (Fig. 1c). The tumor volumes 18 days after treatment were 762.2 ± 132.8 mm^3^ and 251.0 ± 44.65 mm^3^ (mean ± standard deviation) in the control and fisetin treatment groups, respectively (**P ≤ 0.01; Fig. 1d). In addition, immunohistochemistry data from these tumors showed that the proliferation related protein proliferating cell nuclear antigen (PCNA) was significantly reduced in the group with fisetin treatment (Fig. 1e). Western blots revealed that treatment with fisetin decreased expression of cell growth and proliferation related proteins including PCNA, Ki67, and phosphorylated histone H3 (p-H3) (Fig. 1f). Overall, these results demonstrated that fisetin inhibited pancreatic cancer cell proliferation and tumor growth in vivo.

### Fisetin induced apoptosis and autophagy in pancreatic cancer cells

Western blots also showed that fisetin led to an increase in apoptotic proteins including cleaved caspase 3 and cleaved poly (ADP ribose) polymerase (PARP) (Fig. 1f). To further determine whether fisetin-induced cell death was via the apoptotic pathway, we assessed phosphatidylserine translocation using Annexin-V and propidium iodide double-staining. Apoptosis analysis by flow cytometry showed a time-dependent increased in the number of apoptotic cells in the fisetin treatment group (Fig. 1g). The proportion of cells in the lower-right quadrant, which corresponds to early apoptotic cells (Annexin-V positive), were 8.64% ± 0.62% (**P ≤ 0.01) and 21.17% ± 0.26% (****P ≤ 0.0001) after treatment with 200 µM fisetin for 24 and 48 h, respectively. These results demonstrated the ability of fisetin to induce apoptosis in PANC-1 cells.

**Fig. 2:**
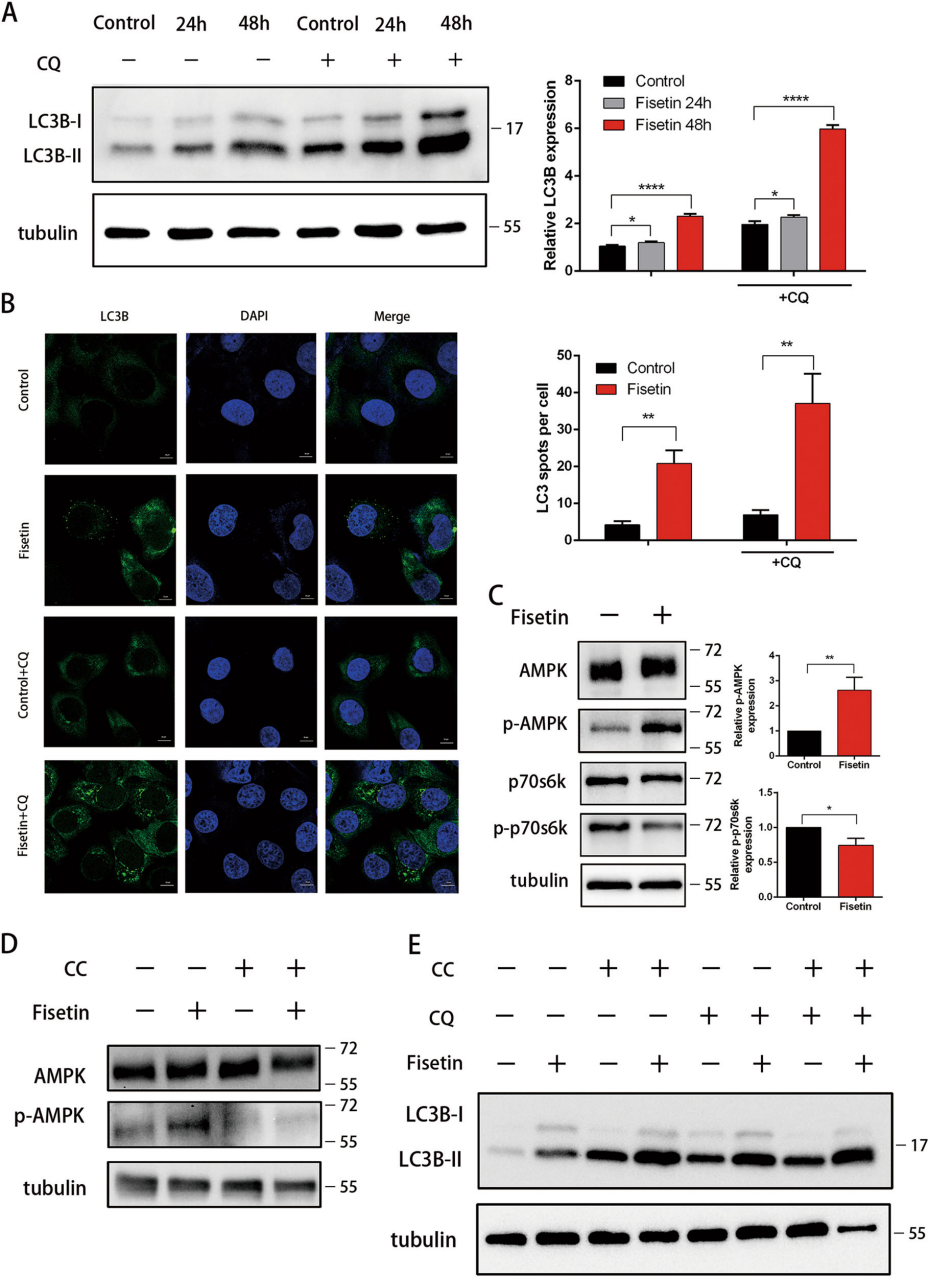
Effect of fisetin on AMPK signaling pathway. To promote autophagosome generation, cells were treated with 20 μM Chloroquine (CQ) for 2 h before collection. **a** Western blotting analyses of LC3B in control (Con) and fisetin treated PANC-1 cells. Cells were treated with fisetin (200 μM) for 24 h and 48 h. Quantitative data of optical band densitometry are shown. Data are presented as mean ± SD (*n* = 5), *P ≤ 0.05, ****P ≤ 0.0001. **b** Analyses of autophagy by observation of the immunofluorescence of LC3B (green)in PANC-1 cells. Cells were treated with fisetin (200 μM) for 48 h. And CQ were used as described previously. The nucleus were counter stained with DAPI (blue). Scale bars 10 μm. Comparative changes in the LC3B spots number in control and treatment groups. The spots were counted by manual scoring. Results are mean ± SD, **P ≤ 0.01. **c** Fisetin-mediated changes in AMPK (*p-AMPK*) and mTOR target protein p70S6K (*p-p70S6K*) in PANC-1 cells. Effect of fisetin on activation of AMPK resulted in inhibition of mTOR target protein. Data are presented as mean ± SD(*n* = 3), *P ≤ 0.05,**P ≤ 0.01. **d** Western blotting analyses of AMPK (*p-AMPK*). Cells were treatment of AMPK inhibitor compound C (CC) (20 μM)for 4 h before collection. Fisetin and CQ were used as described previously. The level of p-AMPK was reduced by CC. **e** Western blotting analyses of LC3B. Fisetin, CC, and CQ were used as described previously

Furthermore, we found that fisetin also induced autophagy in PANC-1 cells (Fig. 2a). To measure autophagic flux, we used chloroquine (CQ) to block lysosomal function and the late degradation stage of autophagy^32^. Western blotting showed that the autophagy marker LC3B was upregulated in fisetin-treated cells (Fig. 2a). Moreover, immunofluorescence showed punctate LC3B-positive autophagic vesicles in PANC-1 cells treated with fisetin for 48 h (Fig. 2b). To further confirm the results, we established PANC-1 cells that stably expressed a tandem mRFP-GFP-LC3 construct. The results further demonstrated that fisetin increased the autophagic flux in PANC-1 cells (Fig. S1C).

### Fisetin stimulated the AMPK pathway in pancreatic cancer cells

The AMPK/mTOR pathway has been well-established as a critical regulator of autophagy^33,34^. To further examine the type of autophagy induced by fisetin, levels of AMPK and its phosphorylated form (p-AMPK) were analyzed by western blot. Moreover, the direct mTOR substrate p70s6k and its phosphorylation status were used as surrogates for mTOR activity^35^. The results showed that p-AMPK levels increased in response to fisetin, whereas p-p70s6k levels decreased (Fig. 2c). Next, PANC-1 cells were treated with the p-AMPK inhibitor compound C (CC) to determine the role of AMPK in autophagy following fisetin treatment. The results showed that p-AMPK levels were reduced by CC. However, western blots showed that LC3B-II levels were not significantly decreased in CC-treated cells (Fig. 2d). Thus, the mechanism of autophagy induction in fisetin-treated PANC-1 cells may be more complex than in other systems.

### Fisetin induced ER stress in pancreatic cancer cells

**Fig. 3:**
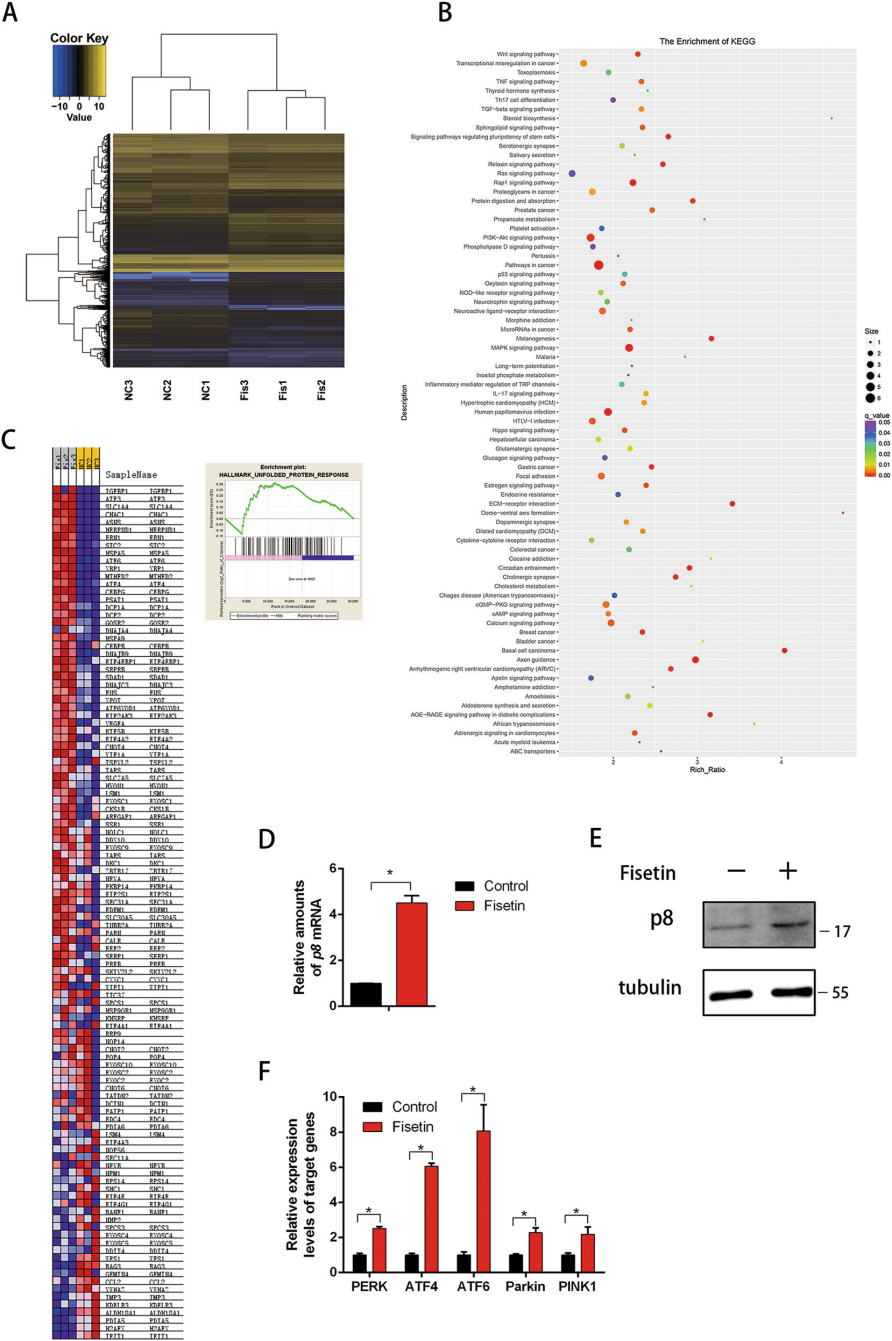
RNA sequencing (RNA-seq) analysis of cells treated with fisetin. **a** Heat map representing unsupervised hierarchical clustering of mRNA expression levels in Pnac-1 cells treated with fisetin (200 μM) for 48 h. Each column represents the indicated sample; each row indicates one mRNA. Yellow and blue indicate high and low expression, respectively. **b** The top 77 enriched KEGG pathways. **c** Gene Set Enrichment Analysis enriched “unfolded protein response (UPR)” pathway. ES of 0.31 and NES of 1.51 (nominal p-value = 0.026, FDR = 0.079). **d** RT-qPCR verification of differentially expressed gene *p8 (NUPR1)* in the RNA-seq of PANC-1 cells. The results are presented as the mean ± SD (*n* = 3), *P ≤ 0.05. **e** Western blot detection of the expression of p8, ɑ-tubulin was used as the internal control. **f** RT-qPCR verification of differentially expressed genes in the RNA-seq enriched pathway of PANC-1 cells. The results are presented as the mean ± SD (*n* = 3), *P ≤ 0.05. Fis fisetin treatment, NC negative control

To explore the molecular details underlying fisetin-induced autophagy in PANC-1 cells, we studied the transcriptome of cells under fisetin treatment using RNA sequencing (RNA-seq) and compared these results with control cells. This analysis identified 2672 differentially regulated genes (*P* ≤ 0.05, *q* ≤ 0.05, fold change ≥ 2), of which 1136 and 1536 were up- and down-regulated, respectively (Fig. 3a, Fig. S2A). Gene Ontology analysis showed that fisetin affected cellular components, biological processes, and molecular functions by regulating 2672 genes in various pathways (Fig. S2A–C). Moreover, the top 77 enriched Kyoto Encyclopedia of Genes and Genomes (KEGG) pathways are presented, and they included various signaling pathways that affect cell fate, such as Wnt, TNF, TGF-β, PI3K/AKT, and p53 signaling pathways (Fig. 3b). Finally, Gene Set Enrichment Analysis revealed “TNF-α signaling via NFκB” as the most significantly associated pathway with an overall enrichment score (ES) of 0.42 and a normalized enrichment score (NES) of 2.22 (nominal *p*-value ≤ 0.001, false discovery rate [FDR] ≤ 0.001)(Fig. S2D,E). Additionally the “p53 pathway” had an ES of 0.29 and an NES of 1.56 (nominal *p*-value = 0.005, FDR = 0.065); p8 was also enriched in this pathway (Fig. S2F–G). Interestingly, we found that the “unfolded protein response (UPR)” pathway had an ES of 0.31 and an NES of 1.51 (nominal *p*-value = 0.026, FDR = 0.079), which suggested that genes related to regulating autophagy were enriched in response to ER stress (Fig. 3c). The accumulation of misfolded proteins can lead to ER stress, which in turn induces an adaptive intracellular response, namely UPR^36^. However, cells undergo apoptosis or autophagy when UPR cannot sufficiently deal with ER stress^37^. Our RNA-seq analysis revealed that p8 mRNA expression was increased, and we further verified this result by quantitative RT-PCR (RT-qPCR) and western blotting (Fig. 3d, e). In addition to p8, RNA-seq also showed that the expression of several key stress sensors in the UPR and autophagy pathways were enhanced in fisetin-treated cells. These included PERK, ATF6, and ATF4, all of which are involved in reticulophagy (ER stress-mediated autophagy)^28,37^, as well as Parkin and PTEN induced kinase 1 (PINK1), which are involved in mitochondrial stress-mediated autophagy^38–40^. We next verified the RNA-seq data by RT-qPCR and the results confirmed the RNA-seq analysis (Fig. 3f)

### Fisetin-induced autophagy was mitochondrial stress-and ER stress-dependent

**Fig. 4:**
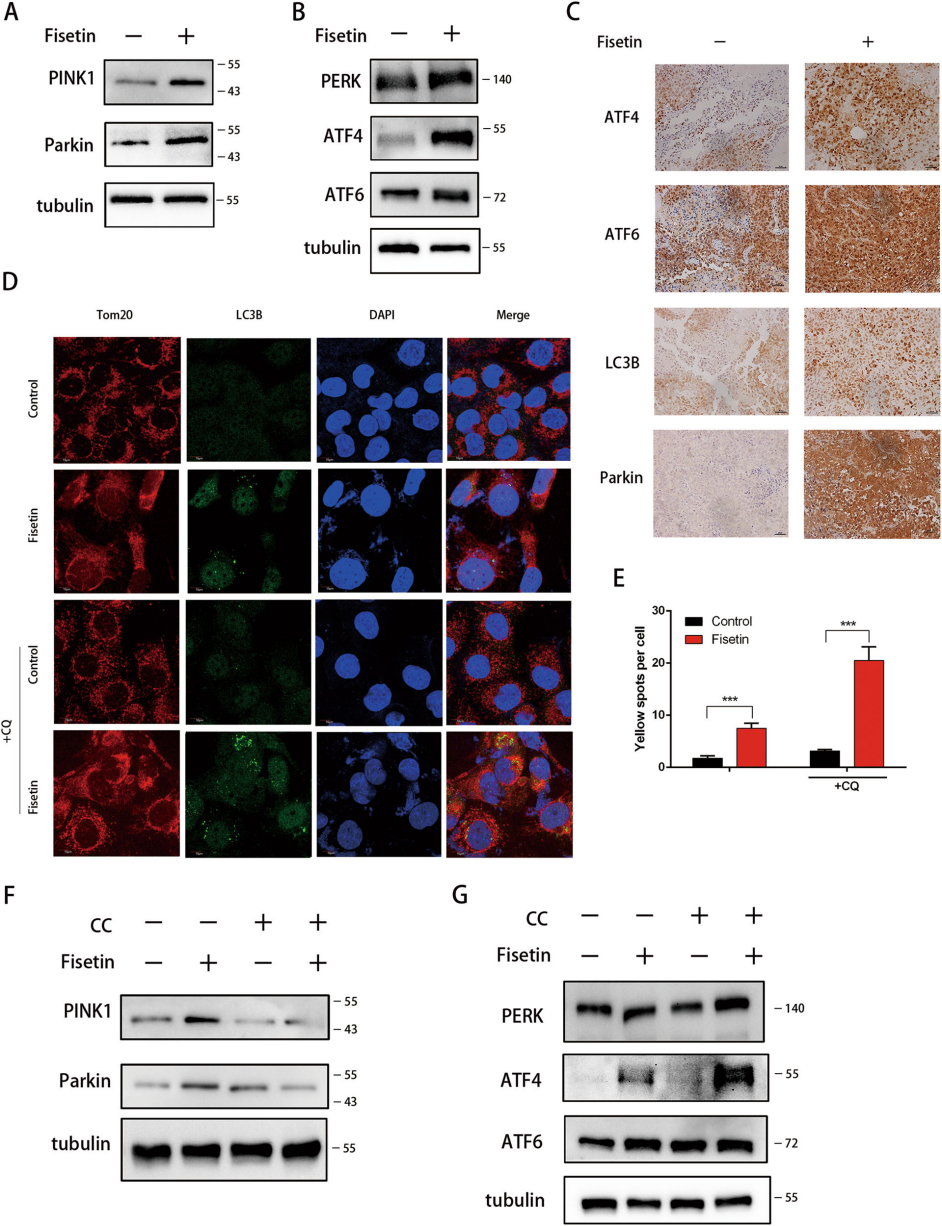
nhibition of AMPK/mTOR signaling did not markedly alleviate the autophagy induced by fisetin. I Cells were treated as described previously. **a** Western blot detection of the expression of PINK1 and Parkin, both which were involved in mitochondrial stress-mediated autophagy. **b** Western blot detection of the expression of PERK, ATF4, and ATF6, both of which were involved in endoplasmic reticulum (ER) stress-mediated autophagy. **c** Expressions of ATF4, ATF6, Parkin, LC3B in control and fisetin treatemt groups of mice were examined by immunohistochemistry. Bar scale 50 μm. **d**, **e** Mitophagy were examined by colocalization analysis of LC3B and Tom20 (a marker of mitochondria). Yellow spots suggested that mitophagy was increased by fisetin. ***P ≤ 0.001. **f**, **g** To inhibit AMPK/mTOR signaling pathway, cells were treated with AMPK inhibitor compound C (CC) (20 μM)for 4 h before collection. Western blot detection showed that expression of PINK1 and Parkin were reduced by CC. However, the expression of PERK, ATF4, ATF6 were not blocked by CC, even the level of ATF4 was increased

To further confirm the results of RNA-seq analysis, we examined these target proteins by western blotting. The data revealed that Parkin and PINK1 levels were increased after treatment (Fig. 4a). Interestingly, the expression of PERK, ATF4, and ATF6 were also upregulated by fisetin (Fig. 4b). Moreover, LC3B, Parkin, ATF4, and ATF6 in vivo expression were also increased by fisetin (Fig. 4c). To examine whether the type of autophagy was mitophagy in response to mitochondrial stress, we measured the mitophagy via colocalization analysis of LC3B and Tom20 (a marker of mitochondria). The immunofluorescence showed colocalized spots (yellow) that suggested mitophagy was increased by fisetin (Fig. 4d, e). To determine whether the expression levels of these proteins were associated with AMPK signaling, we analyzed the levels of these proteins after CC treatment. The results revealed that CC blocked fisetin-induced Parkin and PINK1 expression, while there were no effects on PERK, ATF4, or ATF6 (Fig. 4f, g). These observations suggested that Parkin and PINK1 expression, which are involved in mitophagy, were modulated through an AMPK-dependent pathway, while PERK, ATF4, and ATF6, which are associated with reticulophagy, were not affected by AMPK inhibition.

**Fig. 5:**
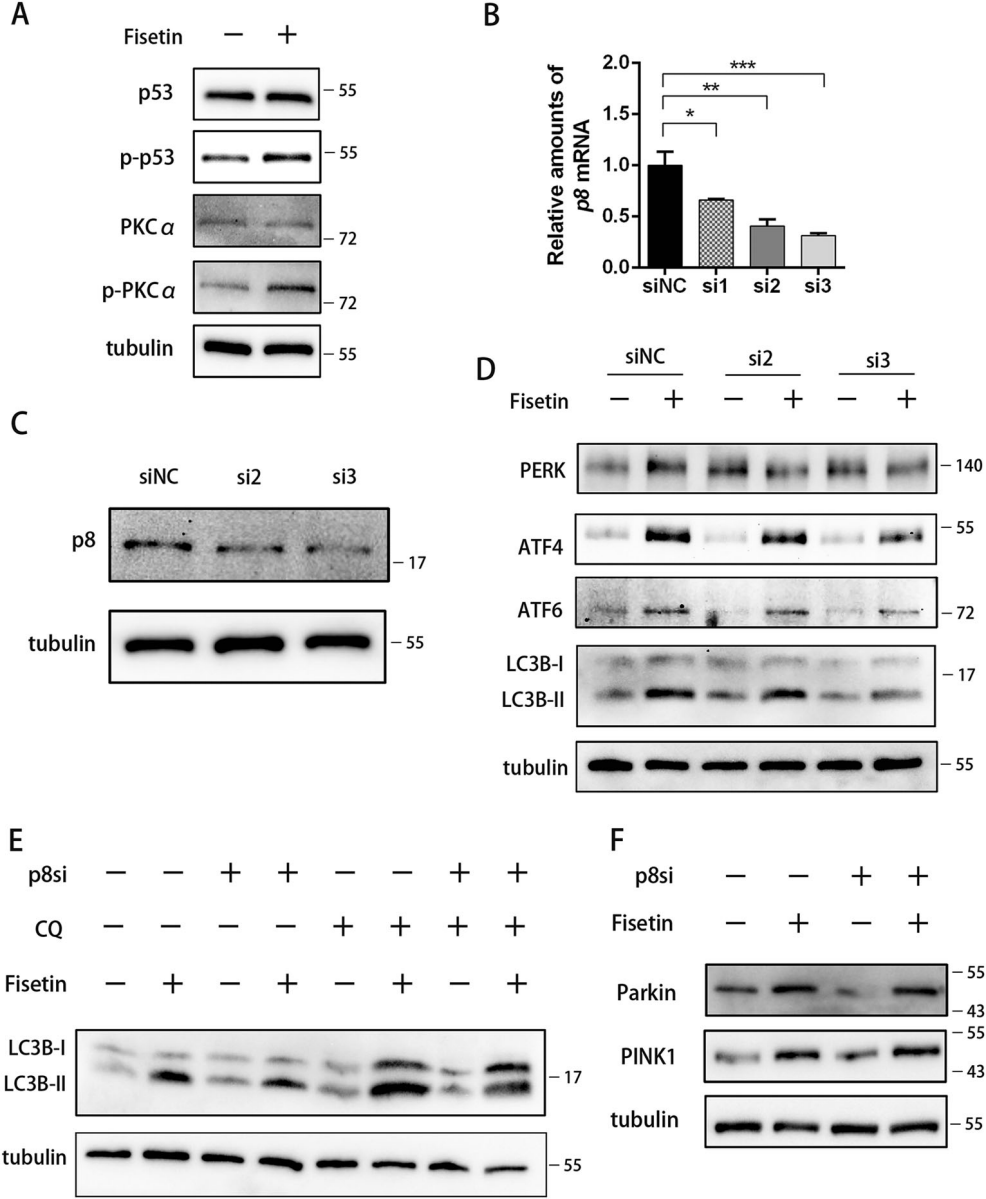
Autophagy was alleviated by silencing p8 in cells treated with fisetin. **a** Western blot detection of the expression of p53, PKCɑ, both of which were involved in p8-dependent pathway. **b** p8-specific siRNAs (100 pM) were transfected into PANC-1 cells with siRNA transfection reagent for 24 h. RT-qPCR of p8 in control (siNC) and p8 interference (p8si1, p8si2, and p8si3) PANC-1 cells with or without 200 μM fisetin treatment for 48 h was performed. The results are presented as the mean ± SD (*n* = 3), *P ≤ 0.05, **P ≤ 0.01,***P ≤ 0.001. **c** Western blotting analyses of p8 in control and p8 interference PANC-1 cells. **d** Western blotting analyses of PERK, ATF4, and ATF6 in control and p8 interference PANC-1 cells with or without fisetin treatment. **e** Western blotting analyses of LC3B in control and p8 interference PANC-1 cells with or without fisetin treatment. To promote autophagosome generation, cells were treated with 20 μM Chloroquine (CQ) for 2 h before collection. **f** Western blotting analyses of Parkin and PINK1 in control and p8 interference PANC-1 cells with or without fisetin treatment

As a critical sensor of cellular stress, p8 is also associated with autophagy in response to ER stress^26,41^. Our previous results^29^ and current RNA-seq analysis (Fig. 3c–f, Fig. S2F–G), led us to focus on p8-regulated autophagy, and the levels of p8’s targets (p53 and PKC-α) by western blot were analyzed to determine the mechanism of fisetin-induced autophagy. The results showed that the phosphorylation of p53 and PKC-α increased, which suggested that p53 and PKC-α were activated following fisetin treatment (Fig. 5a). Although pancreatic cancers carry mutations in the p53 gene, Chien et al. found that loss-of-function mutations of *p53* in pancreatic cancer can be partially circumvented^42^. Interestingly, we found that p21, a downstream target of p53, was also increased by fisetin (Fig. S2H). These results indicated that the loss of function of *p53* was partially circumvented by treatment with fisetin. Our previous studies have shown that p8/p53/PKC-α signaling induces autophagy by activating PERK and ATF6 in response to palmitic acid-mediated stress^29^. To confirm whether the autophagy observed following fisetin treatment was associated with p8, we silenced p8 expression by RNAi; p8 mRNA levels were decreased by 33.8%, 59.5%, and 68.6%, respectively, using three different siRNA sequences (p8si1, p8si2, and p8si3; Fig. 5b). We then picked the two most efficient p8 silencing siRNAs (p8si2 and p8si3) to further test the effects of silencing p8 by western blot analysis. The results demonstrated that expression of p8 was reduced by silencing p8 (Fig. 5c). And we demonstrated that the induction of PERK, ATF4, and ATF6 expression was alleviated by silencing p8 (Fig. 5d). Moreover, LC3B-II expression was significantly reduced in the p8si3 group (Fig. 5d). To measure autophagic flux in p8 silenced cells, we used CQ to block lysosomal function and the late degradation stage of autophagy. The data indicated that autophagic flux was blocked in p8 depleted cells (Fig. 5e). Finally, we studied Parkin and PINK1 levels in p8-silenced cells, and the results showed that p8 did not affect Parkin or PINK1 expression, which suggested that p8 did not influence AMPK-dependent mitophagy (Fig. 5f). Together, these results suggested that p8 regulated PERK, ATF4, and ATF6 expression through the p53/PKC-α signaling pathway to modulate autophagy in response to fisetin-induced ER stress.

### Fisetin-induced autophagy was cytoprotective

**Fig. 6:**
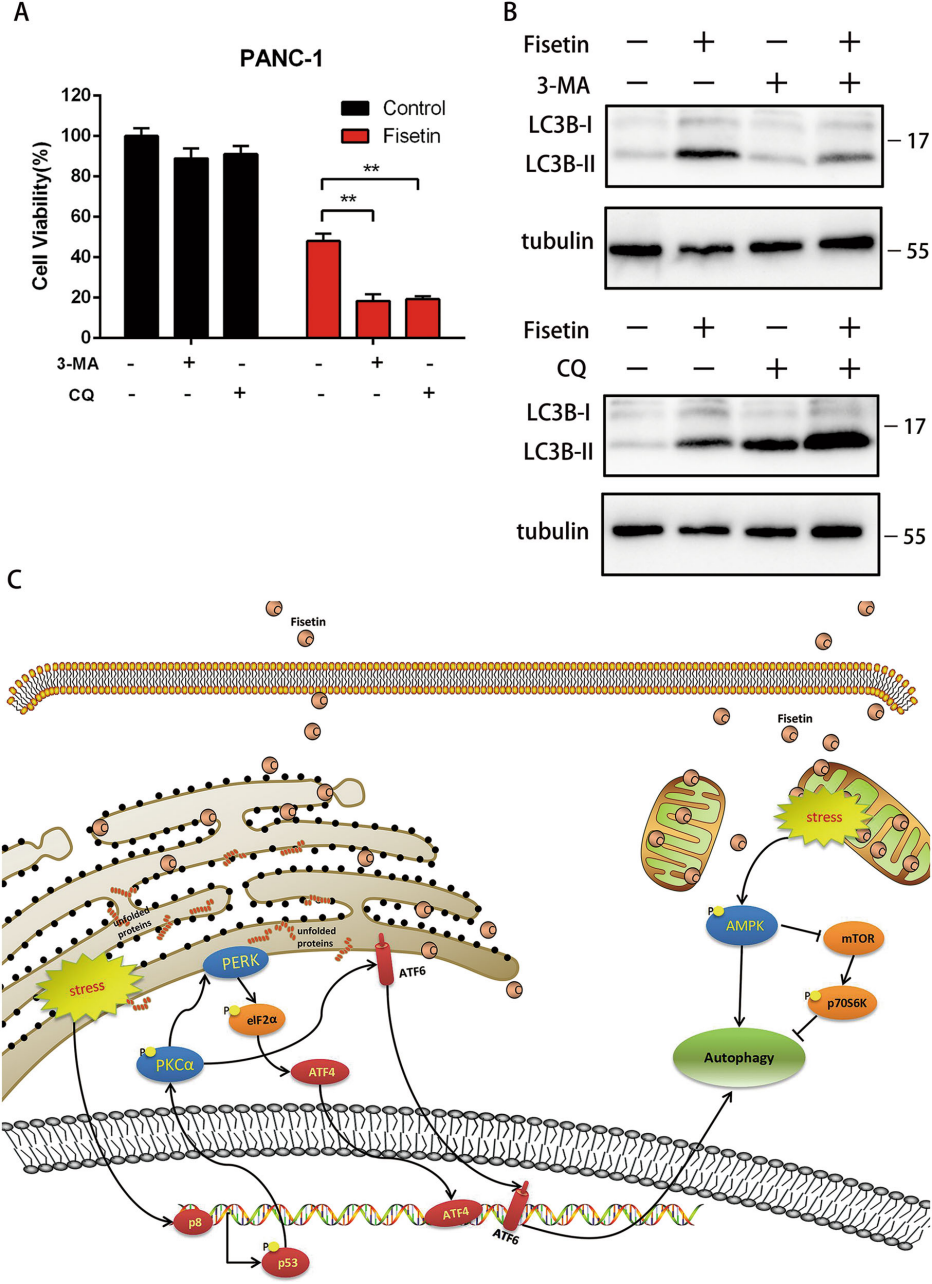
Blocking autophagy enhanced antitumor effect of fisetin. **a** Co-treatment with fisetin and autophagy inhibitors 3-MA or CQ resulted in decreased cell viability. Cell viability was measured by CCK-8 assay. Cells were pre-treated with 3-MA (5 mM) or CQ (40 μM) for 4 h, followed by fisetin (200 μM) for 48 h. Data are presented as mean ± SD; **P ≤ 0.01. **b** Expression of LC3B after co-treatment with fisetin and 3-MA or CQ. **c** Proposed model of the endoplasmic reticulum stress- and mitochondrial stress-dependent autophagy signaling pathway in response to fisetin

Abnormal autophagy is involved in both cell survival and cell death in pancreatic cancer^15,16^. To elucidate the role of fisetin-induced autophagy in PANC-1 cells, we treated cells either with fisetin alone or together with autophagy inhibitor 3-Methyladenine (3-MA) or CQ. Co-treatment with fisetin and 3-MA resulted in decreased viability (18%) compared with the group treated with fisetin alone, which showed a viability of 49% (Fig. 6a, b). Cells treated with fisetin together with CQ also showed decreased viability(19%) (Fig. 6a, b). These data indicated that fisetin induced autophagy was cytoprotective in PANC-1 cells.

## Discussion

Pancreatic cancer is one of the most aggressive cancer types and is associated with poor survival outcomes; the 5-year relative survival rate for patients with all clinical stages of pancreatic cancer combined is 6%^1,2^. This poor survival rate is attributable to late diagnosis and resistance to current chemotherapies. Thus, novel therapeutic approaches are needed to improve outcomes.

Fisetin is a natural flavonoid that has been widely reported to inhibit the proliferation of various cancer cell lines. The antitumor effects of fisetin have been demonstrated to be mediated by altering different signaling pathways in different cellular contexts. As for the effect of fisetin in PDAC, Murtaza et al. showed that fisetin suppressed the growth of pancreatic cancer AsPC-1 cells via inhibiting NF-κB signaling^11^. However, there remains a need for more studies investigating the mechanism of fisetin’s antitumor effects in PDAC. In other cancers types, such as prostate and colon cancer, fisetin stimulated different pathways to induce apoptosis^8,9^. Fisetin also induced autophagy in prostate cancer and melanoma cells^20,43^. The relationship between apoptosis and autophagy is complicated but important for deciding cell fate. Only a few studies have observed autophagy in cancer cells following fisetin treatment. Suh et al. found that fisetin-induced autophagic cell death in PC-3 prostate cancer cells was initiated through the AMPK/mTOR-dependent pathway^20^. Utomo et al. found that inhibition of mTOR in pancreatic cancer induced autophagy and was associated with cancer cell death^44^. Interestingly, another study indicated that fisetin induced transient autophagy in response to ER stress via an AMPK-independent pathway. Clearly, more studies are needed to determine the role of autophagy in fisetin-treated cancer cells.

In this study we dissected the antitumor effects of fisetin in human pancreatic cancer PANC-1 cells in vitro and in vivo, and primarily focused on the role of fisetin-induced autophagy. Our results showed that fisetin inhibited PANC-1 cell proliferation in vitro using a 2D culture system and in vivo using xenograft tumor models. Interestingly, we found that low concentrations of fisetin(25, 50 µM) had no significant antitumor effect. Emerging studies showed that food-derived polyphenols such as genistein, exhibit a dual, dose-dependent effect on cancer cells^45^. It is also of note that PANC-1 is one of the aggressive and most resistant pancreatic cancer cell lines, and is more resistant to gemcitabine than pancreatic cancer AsPC-1 and BxPC-3 cells^46,47^. Thus, to further explore the effect of fisetin, we used a high concentration (200 µM), which led to about 50% cell viability in PANC-1 cells. Additionally, we showed that fisetin induced apoptosis, autophagy and the AMPK/mTOR pathway in PANC-1 cells.

To further determine the mechanism of autophagy, we used CC to inhibit the AMPK/mTOR pathway. However, we found that autophagy was not significantly decreased by CC. Thus, we studied the transcriptome of fisetin-treated cells using RNA-seq. The Gene Set Enrichment Analysis results showed that genes related to the “UPR” pathway were upregulated in fisetin-treated cells. The UPR is an intracellular adaptive response to cope with ER stress. When UPR cannot deal with the ER stress, cell fate will be determined by apoptosis and autophagy^36,37^. Our previous studies and other reports have indicated that p8 is a key stress sensor that plays a critical role in ER stress-induced autophagy^26,29,41^. Interestingly, a previous study indicated that fisetin induced ER stress and transient autophagy in human melanoma cells^43^. Thus, we hypothesized that there could be more than one form of autophagy. We analyzed several key proteins that are involved in ER stress, including PERK, ATF4, and ATF6, and the hallmark proteins of mitophagy, Parkin and PINK1. Expression levels of these proteins were increased by fisetin treatment, but only Parkin and PINK1 were reduced by the AMPK inhibitor CC, which suggested that AMPK was not involved in ER stress-mediated autophagy signaling. Thus, we next analyzed levels of p8, which regulates PERK and ATF6 in response to palmitic acid-mediated stress^29^. We found that the levels of p8 and its targets p53 and PKC-α were increased in fisetin-treated cells. Although PANC-1 cells carry a mutated *p53* gene, the lack of function of p53 was partially circumvented by fisetin. Next, we silenced p8 to determine the relationship between p8 and PERK, ATF4, ATF6, Parkin and PINK1 in fisetin-treated cells. The results showed that p8 regulated PERK, ATF4, and ATF6 expression but did not affect Parkin or PINK1. Interestingly, fisetin combined with CC did not reduce LC3B, but increased ATF4 and ATF6. Thus, we conclude that there might be crosstalk between the AMPK/mTOR signaling pathway and p8-dependent autophagy pathway, which needs further investigation.

Autophagy is not only recognized as a type of programmed cell death, but also associated with cell survival. Thus, we treated cells either with fisetin alone or together with autophagy inhibitor 3-MA or CQ to explore the role of autophagy induced by fisetin in PANC-1 cells. We discovered that fisetin induced autophagy was cytoprotective. Autophagy inhibitors CQ and 3-MA can augment the antitumor effect of fisetin in PANC-1 cells.

In conclusion, we have demonstrated that fisetin-treated PANC-1 cells show increased autophagy, which is mediated by p8 through the p53/PKC-α pathway, which in turn affects PERK, ATF4, and ATF6 levels, and is also involved in mitophagy. We revealed that the autophagy induced by fisetin in PANC-1 cells is cytoprotective. Combination with autophagy inhibitors can significantly strengthen the effect of fisetin in PANC-1 cells. The mechanisms that underlie the circumvention of mutated *p53* and the crosstalk between AMPK/mTOR signaling and p8-dependent pathways need to be further studied. These findings provide the basis for an in-depth study of autophagy in pancreatic cancer.

## Materials and methods

### Cell culture

Human pancreatic cancer PANC-1, BxPC-3 cells were kindly provided by Stem Cell Bank, Chinese Academy of Science (SCSP-535, TCHu 12, China). Human normal prancreatic duct hTERT-HPNE cells were provided by Cobioer (CBP60875, China). PANC-1, BxPC-3 and hTERT-HPNE cells were authenticated by short tandem repeat profiling at the Stem Cell Bank, Chinese Academy of Science, and comparison to the published profile.And PANC-1, BxPC-3 and hTERT-HPNE cells were last tested by short tandem repeat profiling on May and November 2018. These cells were cultured in DMEM (Gibco) or 1640 (Gibco) supplemented with 10% FBS, 1% penicillin/streptomycin, and were growth at 37 °C in a humidified atmosphere consisting of 5% CO_2_.

### Reagents and antibodies

Fisetin was purchased from Selleck (catalog.S2298), compound C (catalog.HY-13418A) and 3-Methyladenine (catalog.HY-19312) were obtained from MedChemExpress and Chloroquine (CQ) was purchased from Sigma. The primary antibodies used in this study were as follows: polyclonal anti-p-AMPK (Epitomics, Burlingame, CA, catalog no.3930-1), polyclonal anti-p-p70s6k (Cell Signaling Technology, Leiden, The Netherlands, catalog no.9205 s), polyclonal anti-AMPK (Cell Signaling Technology, catalog no.2532), polyclonal anti-p70s6k (Epitomics, catalog no.1175-1), polyclonal anti-ɑ-tubulin (Sigma, catalog no.T6199), polyclonal anti-p8 (Abcam, Cambridge, UK, catalog no.ab46889; Santa Cruz Biotechnology, catalog no.sc-30184), monoclonal anti-LC3B (Sigma, catalog no.L7543; Cell Signaling Technology, catalog no.3868), polyclonal anti-PERK (Cell Signaling Technology, catalog no.5683), polyclonal anti-ATF6 (Abcam, catalog no.ab37149), polyclonal anti-p-PKCɑ (Millipore, Nottingham, UK, catalog no.06-822), polyclonal anti-PKCɑ (Cell Signaling Technology, catalog no.2056), polyclonal anti-p-p53 (Cell Signaling Technology, catalog no.9284P), polyclonal anti-p53 (Epitomics, catalog no.1026-1), monoclonal anti-PINK1 (Abcam, catalog no.ab216144), and monoclonal anti-Parkin (Abcam, catalog no.ab179812), polyclonal anti-Tom20 (Santa Cruz Biotechnology, catalog no.sc-17764), monoclonal anti-PCNA (Abcam, catalog no.ab92552), monoclonal anti-phosphorylated histone H3 (Ser10) (Abcam, catalog no.ab177218), monoclonal anti-Ki67 (Abcam, catalog no.ab92742), monoclonal anti-cleaved-casepase3 (Cell Signaling Technology, catalog no.9664), monoclonal anti-cleaved -PARP (Cell Signaling Technology, catalog no.5625).

### Real-time cell analysis by xCELLigence

Cell growth was measured at sparse density in real time using a well-described system (xCELLigence, Roche). Growth curve assays were performed in triplicate, in real time, with the xCELLigence System (Roche Applied Science). xCELLigence plates were seeded with 5000–10,000 cells per well and cultured with different dose of fisetin (0, 100, 200, 400 μM), and growth reported as Cell Index which reflects a consistent, logarithmic relationship to cell number. All data is presented as the mean normalized Cell Index ± SD over time.

### Cell viability assay

Cells were seeded and cultured with fisetin (0, 25, 50, 100, 200, 400 μM) for 24, 48 h. Culture media were replaced with 1% CCK-8 solution and incubated at 37 °C for 2 h. After brief incubation, the absorbance of the solution was measured in a microplate reader.

### Xenograft mouse model

Animal experiments were carried out in compliance with the institutional guidelines for the care and use of experimental animals.Ten female BALB/c nude mice were obtained from the Chinese Academy of Sciences Shanghai Experimental Animal Center. Subcutaneous xenograft mouse model were established in 4- to 6-week-old mice by injection of 1 × 10^6^ PANC-1-Luciferase cells in the shoulders via a left subcostal incision under 3% isoflurane anesthesia. Mice were divided into two groups with five mice per group. Control animals were treated with 0.1 ml DMSO via intraperitoneal injection. Treatment group mice were treated with 0.1 ml Fisetin(300 mg/kg body weight i.p.) every other day. Tumor volume was calculated by the formula 0.52 × length × width^2^.

### Bioluminescence imaging

Briefly, for the detection of bioluminescence, mice were anesthetized with 3% isoflurane. Then mice were injected intraperitoneally with 200 μl (15 mg/ml)of D-luciferin. Tumors were imaged after 15 min of biodistribution, with an IVIS Lumina Series III (Perkin Elmer, USA). The same region of interest (ROI) was applied on all the bioluminescent tumors. Data were expressed as average radiance (photons/second/square centimeter/steradian), which is a calibrated measurement of photon emission.

### Transfection and siRNA

P8 siRNAs (Ribobio, China) were used to knock down p8 gene expression. The p8 and control siRNA (100 pM) were transfected into PANC-1 cells using siRNA transfection reagent (Santa Cruz Biotechnology) 24 h before fisetin treatment. The cells were collected after fisetin treatment for 48 h. The Ubi-MCS-Luc-IRES-Puromycin vector were used to construct PANC-1-Luciferase cells. Cells were transfected using Lipofectamine 3000(Invitrogen, USA) and selected for puromycin resistance. MRFP-GFP-LC3 adenoviral vectors were purchased from HanBio Technology (Shanghai, China). PANC-1 cells were plated in 6-well plates and allowed to reach 50–70% confluence at the time of transfection. The GFP fluorescent signal could be quenched under the acidic condition inside 'the lysosome, while the mRFP fluorescent signal has no significant change under the acidic condition. Thus, autolysosomes are shown as red spots (RFP + GFP − ), while autophagosomes are shown as yellow spots (RFP + GFP + ) in mergered images. Autophagic flux is increased when both yellow and red spots are increased, while autophagic flux is blocked when both red and yellow spots are decreased, or when only yellow spots are increased without changes of red spots in cells^48^.

### Detection of apoptosis

Levels of cell apoptosis was tested using BD PE Annexin V Apoptosis Detection Kit I (559763). Cells were washed cells twice with cold PBS and then resuspend cells in 1x Binding Buffer at a concentration of 1 × 10^6^ cells/ml. Then transfer 100 μl of the solution (1 × 10^5^ cells) to a 5 ml culture tube and add 5 μl of PE Annexin V and 5 μl 7-AAD. Gently vortex the cells and incubate for 15 min at RT (25 °C) in the dark. Add 400 μl of 1x Binding Buffer to each tube. Analyze by flow cytometry within 1 h.

### Immunofluorescence via confocal microscopy

PANC-1 cells were fixed with 4% paraformaldehyde at 4 °C overnight and then washed with PBS in 5 min for three times. Fixed cells were blocked in antibody dilution buffer (PBS containing 0.25% Triton X-100 and 1% bovine serum albumin), and all subsequent staining was performed in the same buffer. The cells were labeled with primary antibody for 2 h and subsequently with Alexa Fluor 488-conjugated donkey anti-mouse secondary antibody (Invitrogen Life Technologies) for 1.5 h. The nucleus was stained by incubation with DAPI for 15 min at room temperature. Cells were scanned using a ZEISS LSM 800 confocal microscope (Zeiss, Jena, TH, GER) equipped with a 63 × objective.

### Western blotting analysis

Cells were lysed with RIPA buffer (Sigma, St.Louis, MO, USA) containing a mixture of protease inhibitor cocktail kit (Thermo, Rockford, IL, USA). Then the lysates were cleared by centrifugation and the concentrations of proteins were measured by BCA protein assay kit (Pierce, USA).Each total protein sample (4 µg) was separated on SDS-PAGE gels and then transferred to PVDF membranes (Bio-Rad). After incubation of the membranes with primary antibodies at 4 °C overnight, the samples were incubated with the secondary antibodies conjugated with horseradish peroxidase for for 1 h at room temperature.The blots were visualized using a ChemiDoc™ Touch Imaging System (Bio-Rad).

### Immunohistochemistry

Immunohistochemical staining was performed using the standard streptavidin-biotin-peroxidase complex method. Briefly, paraffin-embedded tissue sections were dewaxed and rehydrated through an alcohol series, and endogenous peroxidase activities were blocked. Slides were incubated overnight at 4 °C with primary antibodies. Samples incubated with PBS instead of primary antibody were used as the negative control. Proteins were detected using secondary antibodies and counter stained in Gill’s hematoxylin, then dehydrated in an ascending methanol series prior to clearing in xylene and mounting under a coverslip. The sections were observed under an Olympus CX31 microscope (Olympus, Tokyo, Japan).

### Real-Time qPCR

Real-Time qPCR was performed to validate the expression of significantly altered levels of mRNAs. Total RNA was isolated and purified using an miRNeasy Mini Kit (Qiagen, USA) following the manufacturer’s instructions. The extracted RNA was quantified by measuring the absorbance at 260 nm with a PuXin UV/visible spectrophotometer TU-1810). Total RNA (1 μg) was reverse transcribed in a 10 μl reaction containing oligo (dT) primers and M-MLV Reverse Transcriptase (TaKaRa, 2641 A). All real-time PCR analyses were performed using gene-specific primers and the LightCycler® 480 real-time PCR system (Roche) using SYBR Premix Ex TaqTM (TaKaRa, RR420A). The relative amounts of each mRNA were analyzed using the comparative CT method, as described by Schmittgen and Livak^49^. All data are expressed as the mean ± SE of *n* = 3 independent repeats. All statistical analyses were performed using a two-tailed, paired Student’s *t*-test, and *P* ≤ 0.01 was considered significant. Primer sequences were listed in Table. S1.

### RNA sequencing analysis

Cells were treated with Fisetin or DMSO for 48 h. Then, total RNA was extracted from cells using TRIzol (Invitrogen). Library construction and sequencing were performed by Annoroad Gene Technology (Beijing, China). The libraries were sequenced on an Illumina HiSeq 2500 platform and 100-bp paired-end reads were generated. All primary data in RNA sequencing (RNA-seq) analysis have been uploaded to the Gene Expression Omnibus.

### Gene ontology, Kyoto encyclopedia of genes and genomes, and gene set enrichment analyses

The RNA-Seq data were transformed into log2 scale, and Gene Ontology (GO, www.geneontology.org/) analysis, Kyoto Encyclopedia of Genes and Genomes (KEGG, www.genome.jp/kegg/) analysis, and Gene Set Enrichment Analysis (GSEA, http://gsea.org/), were performed to identify the functions and associated enriched pathways of differentially expressed mRNAs.

### Ethics statement

The research protocol was reviewed and approved by the Research Ethics Committee of Sir Run Run Shaw Hospital, School of Medicine, Zhejiang University. Animal experiments were approved and performed in accordance with the institutional guidelines for animal care of animal ethics committee of Zhejiang University.

### Statistical analysis of the data

The data were compiled with the software package SPSS 23.0(SPSS Inc.; Chicago, IL, USA) and GraphPad Prism 6 (San Diego, CA) software. Data were presented as mean ± standard deviation. The Student’s *t*-test was used to compare the means of groups and *P-*values ≤ 0.05 were considered significant.

### Supplementary information

Fig. S1

Fig. S2

Supplementary figure legends

Supplementary Table. S1
