# Correction: Fisetin induces autophagy in pancreatic cancer cells via endoplasmic reticulum stress- and mitochondrial stress-dependent pathways

**DOI:** 10.1038/s41419-023-06399-3

**Published:** 2024-01-29

**Authors:** Shengnan Jia, Xiaodong Xu, Senhao Zhou, Yan Chen, Guoping Ding, Liping Cao

**Affiliations:** 1 Department of General Surgery, Sir Run Run Shaw Hospital, School of Medicine, Zhejiang University, 310000 Hangzhou, Zhejiang China; 2 Department of General Surgery, Huzhou Hospital, Zhejiang University School of Medicine, 313003 Huzhou, Zhejiang China; 3 Innovation Center for Minimally Invasive Technique and Device, Zhejiang University, 310000 Hangzhou, Zhejiang China

Correction to: *Cell Death and Disease* 10.1038/s41419-019-1366-y, published online 13 February 2019

In this article, the flow cytometry pictures of the control and 24 h in Fig. 1G were overlapped due to pasting errors.

The original figure is:
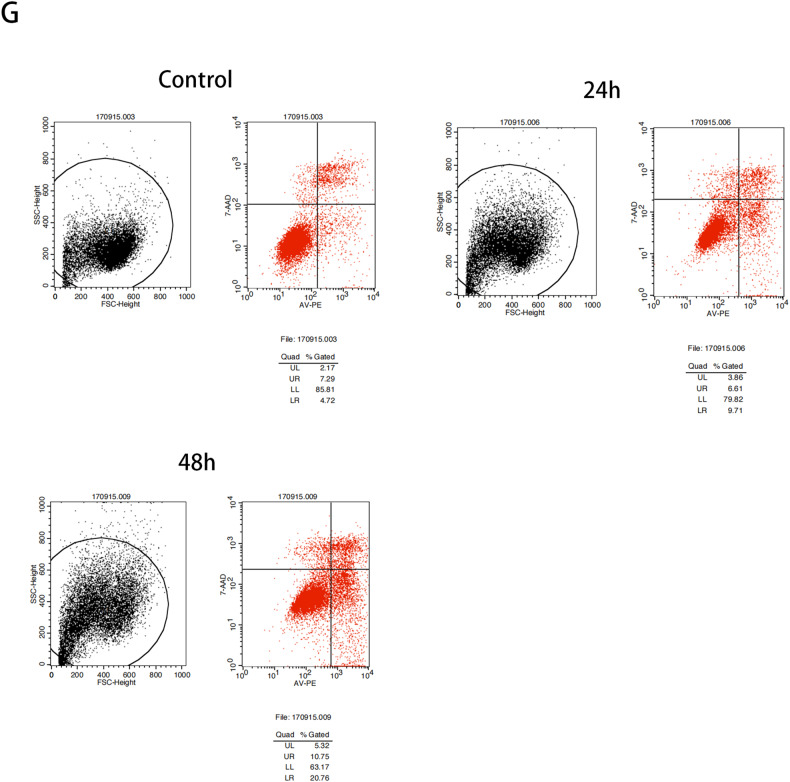


The figure should be read:
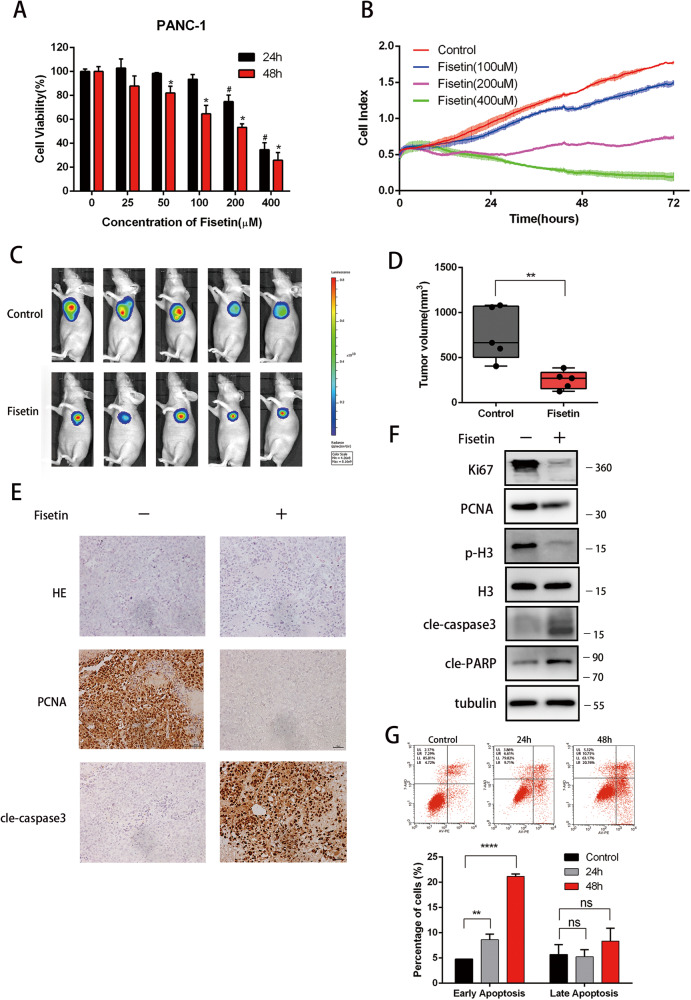


The original article has been corrected.

