# Supplementary figures and images for: Fisetin induces autophagy in pancreatic cancer cells via endoplasmic reticulum stress- and mitochondrial stress-dependent pathways
Source: Cell Death Dis. 2019 Feb 13;10(2):142. doi: 10.1038/s41419-019-1366-y (PMC6374379; doi:10.1038/s41419-019-1366-y)

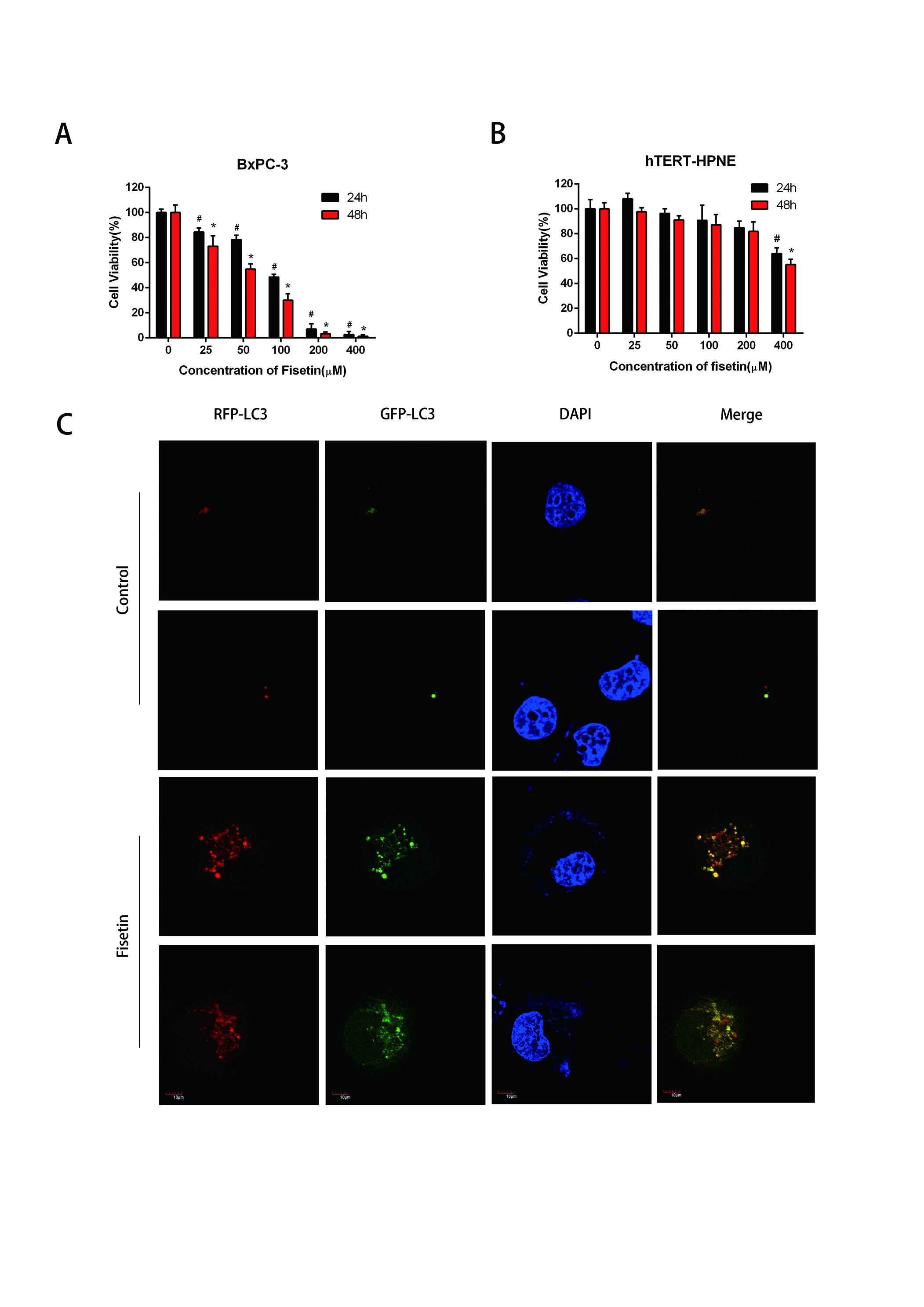

Supplement: Supplementary file 1 — Fig. S1 [file 41419_2019_1366_MOESM1_ESM.tif]

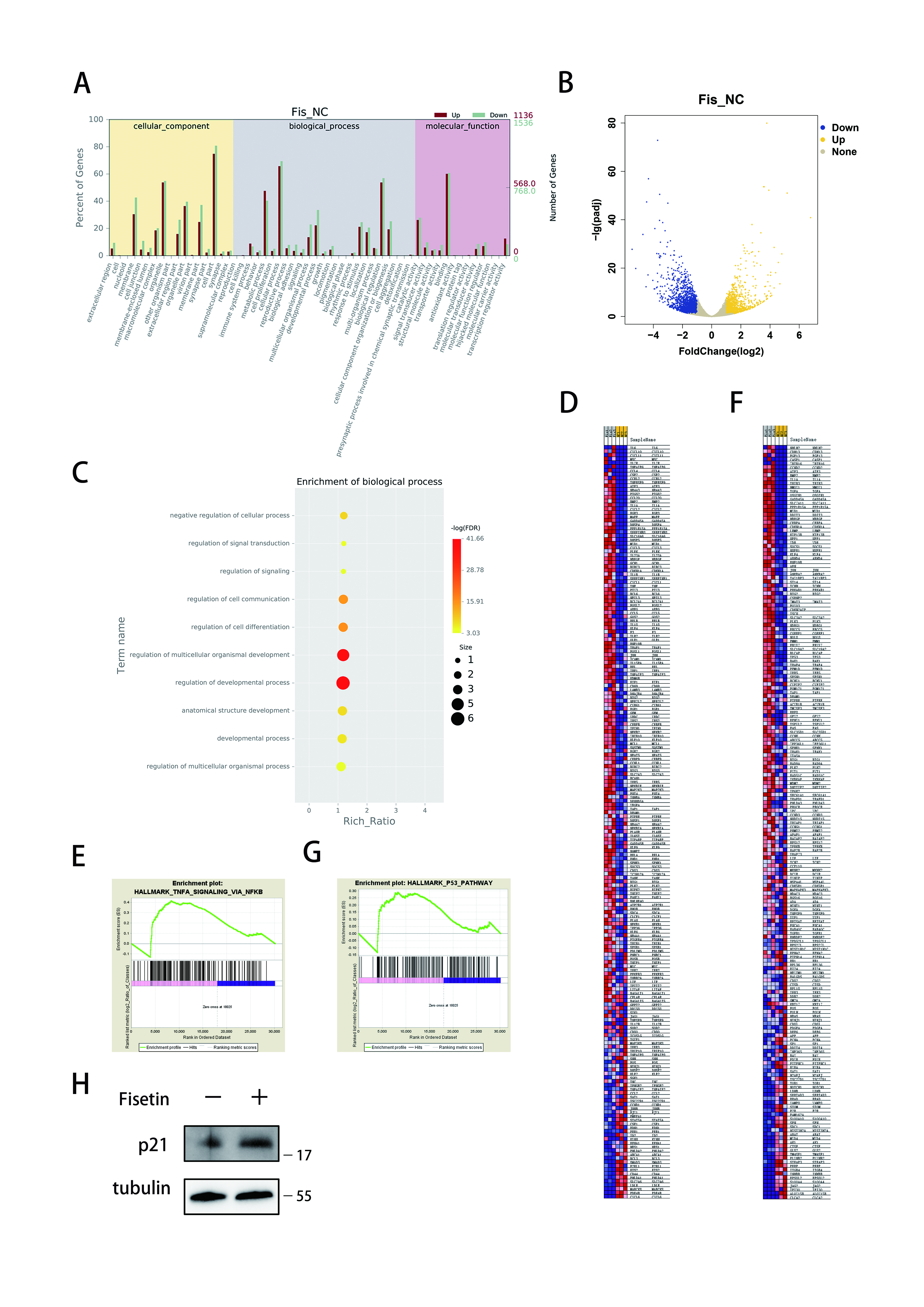

Supplement: Supplementary file 2 — Fig. S2 [file 41419_2019_1366_MOESM2_ESM.tif]
