# Supplementary material for: Fisetin induces autophagy in pancreatic cancer cells via endoplasmic reticulum stress- and mitochondrial stress-dependent pathways
Source: Cell Death Dis. 2019 Feb 13;10(2):142. doi: 10.1038/s41419-019-1366-y (PMC6374379; doi:10.1038/s41419-019-1366-y)
Supplement: Supplementary file 3 — Supplementary figure legends [file 41419_2019_1366_MOESM3_ESM.doc]

**Fig. S1. A-B** Cell viability of pancreatic cancer BxPC-3 and normal prancreatic duct hTERT-HPNE cells was measured by CCK-8 assay. Cells were treated with fisetin (0, 25, 50, 100, 200 and 400 μM) for 24 h and 48 h, respectively. Data are presented as mean ± SD; **P*≤*0.05*, #*P*≤*0.05*. **C** PANC-1 cells that expressed mRFP-EGFP-LC3 fusion protein were treated with fisetin. Confocal microscopic analysis is shown (1200 x magnification). Autolysosomes are shown as red spots (RFP+GFP−), while autophagosomes were shown as yellow spots (RFP+GFP+) in mergered images. Autophagic flux was increased when both yellow and red spots were increased in cells. Bar scale, 10 μm.

**Fig. S2.** RNA sequencing(RNA-seq) analysis of cells treated with fisetin. #Fis, fisetin treatment; NC, negative control. **A-B** Gene Ontology analysis about changes of 2672 genes related with cellular components, biological processes, and molecular functions in Pnac-1 cells treated with fisetin (200μM) for 48h. **C** Gene Ontology analysis enriched biological processes. **D-E** Gene Set Enrichment Analysis enriched “TNF-α signaling via NFκB” pathway. Enrichment score (ES) of 0.42 and a normalized enrichment score (NES) of 2.22 (nominal p-value≤0.001, false discovery rate (FDR)≤0.001). Enrichment plots of “TNF-α signaling via NFκB” pathway revealed the profile of the running ES score & positions of GeneSet Members on the rank ordered list. **F-G** Gene Set Enrichment Analysis enriched “p53 pathway”. ES of 0.29 and an NES of 1.56 (nominal p-value=0.005, FDR=0.065). Enrichment plots revealed the profile of the running ES score & positions of GeneSet Members on the rank ordered list. **H** Western blot detection of the expression of p21.
