# Supplementary material for: Fisetin induces autophagy in pancreatic cancer cells via endoplasmic reticulum stress- and mitochondrial stress-dependent pathways
Source: Cell Death Dis. 2019 Feb 13;10(2):142. doi: 10.1038/s41419-019-1366-y (PMC6374379; doi:10.1038/s41419-019-1366-y)
Supplement: Supplementary file 4 — Supplementary Table. S1 [file 41419_2019_1366_MOESM4_ESM.doc]

**Supplementary Table.S1.** Primer sequences used for real time PCR.

| Genes | Forward primers | Reverse primers |
| --- | --- | --- |
| Nupr1(p8) | GGTCGCACCAAGAGAGAAGC | GGCCTCATCTCCAGCTCTGT |
| ATF4 | TCCAACAACAGCAAGGAGGA | CCAACGTGGTCAGAAGGTCA |
| ATF6 | TCATCTCCTCGGTCAGTGGAC | AGTGGCTCCGGTGAAGAGAG |
| PERK | ATGATCATTCCTTCCCTGGAT | AGTCAGAGATTTTCCTCCAACC |
| Parkin | GCTGACCAGTTGCGTGTGAT | TCTCCACGGTCTCTGCACAA |
| GAPDH | CGGAGTCAACGGATTTGGTCGTAT | AGCCTTCTCCATGGTGGTGAAGAC |
